# Health professionals’ willingness to pay and associated factors for human papilloma virus vaccination to prevent cervical cancer at College of Medicine and Health Sciences University of Gondar, Northwest Ethiopia

**DOI:** 10.1186/s13104-019-4085-7

**Published:** 2019-01-24

**Authors:** Abebe Ayinalem Tarekegn, Ayenew Engida Yismaw

**Affiliations:** 1 Department of Health Service Management and Health Economics, Institute of Public Health and Department of Human Anatomy, University of Gondar, Gondar, Ethiopia; 2 School of Midwifery, College of Medicine and Health Sciences, University of Gondar, Gondar, Ethiopia

**Keywords:** Cervical cancer, Human papilloma virus, Willingness to pay, University of Gondar

## Abstract

**Objective:**

Preferences of health professionals’ for human papilloma virus vaccines was measured by monetary value through willingness to pay (WTP) approach that could help policy makers set priorities among alternative cervical cancer prevention methods in poor countries. The objective of this study was to assess the female health professionals’ willingness to accept and pay, and associated factors for human papilloma virus vaccination at College of Medicine and Health Sciences, University of Gondar, Northwest Ethiopia.

**Results:**

The majority (85.97%) of health professionals’ were willing to pay for Human papilloma virus vaccine. On the average, the respondents were willing to pay 231.34 ETB (US$8.50) per human papilloma virus vaccination service. Age, educational status, knowledge about cervical cancer and its risk factors and monthly income were identified as significant factors to WTP for human papilloma virus vaccination. Policy makers shall consider human papilloma virus vaccine to prevent cervical cancer maintain health of women’s and do more on rising awareness of individuals about cervical cancer and its risk factors.

## Introduction

Cervical cancer is a chronic disease that arises due to the abnormal growth of cells from lower part of uterus which projects to the vagina [1]. Among the causes of cervical cancer, human papilloma virus which account for over 99% of all cases and around 73% is due to HPV16/18 human papilloma viruses [2]. Type of human papilloma virus (HPV), immune suppression, multi-sexual partner, HPV co-infection with other sexually transmitted agents human immunodeficiency virus, multiparity and family history are risk factors which increases persistence of human papilloma virus infection of cervical to develop cervical cancer [3–5].

Every year, about 500,000 cervical cancer cases are diagnosed [6] and Sub-Saharan African countries account 22% of all cases in the globe [7]. Cervical cancer causes 266,000 women to die each year in the world [3]. In Eastern Africa, cervical cancer is estimated to be 42.7 cases per 100,000 women and cervical cancer causes death of 35 per 100,000 women [3, 4, 8, 9]. The incidence and death rate of cervical cancer was 26.4 and 18.4 per 100,000 women’s in Ethiopia, respectively [10].

Loss of 1.5% of the world’s gross domestic product (GDP) associated with cancer due to mortality and incapacity without including direct medical cost of the treatment. In low-income countries burden of cancer was associated with sickness and death of mouth and throat cancer, cervix cancer, and breast cancer which were costing 1.3US$ billion, 1.3US$ billion and 1.1US$ billion respectively, in 2008 [11].

Many countries in the world adopt human papilloma virus vaccine (HPV) as a strategy to overcome health and economic challenges which comes with cervical cancer [3].

The government of Ethiopia had launched cervical cancer screening program in different site with the help of donors since 2013 and has a plan to introduce HPV in the country so as to prevent the incidence and negative impact of cervical cancer. To introduce HPV vaccine to Ethiopia, there is a need to study on willingness to accept and pay for HPV vaccine. Therefore this study was aimed to assess the acceptance rate of female health professionals for HPV vaccine and its benefit interim of money through willingness to pay approach.

Besides, policy makers may know and look for optional strategies to tackle cervical cancer.

## Main text

### Methods and material

#### Study design and setting

Institutional based cross sectional study was conducted to investigate factors associated with willingness to accept and pay for HPV at the College of Medicine and Health Sciences, University of Gondar. The University of Gondar is a higher institution in Ethiopia located in northwest part of the country. The study was conducted from March 1st to April 30th, 2018.

#### Study population and sampling

All female health professionals who were working at CMHS were the study population.

The sample size of the study was calculated by using single population proportion formula for willingness to pay using the Epi Info™ 7 Stat Calc program with the assumption of 50% willingness to pay (p), 95% confidence level, 5% margin of error, and 10% response rate. The final sample size of the study was 423 female health professionals.

The study participants were selected randomly by lottery method from the list of study population.

#### Data collection procedures

An interviewer administered questionnaire was used to collect the data. Double-bounded dichotomous choice variant of the Contingent Valuation method and hypothetical scenario about HPV which was used to estimate the benefit of the program by eliciting the WTP of female health professionals. This method was used to estimate the benefit of the program in terms of monetary unit. The study participants were asked the maximum amount they were willing to pay for the service they got per visit.

#### Operational definitions

*Willingness to pay* is the maximum price the study participant will definitely be willing to buy per unit of a service.

*Contingent value method* is an approach where the study participant was first asked whether they would be willing to pay 200 ETB per service to elicit the preference and then the question was repeated using a higher or lower bid value depending on the response to the first question until reaching to the final maximum amount the study participant will definitely buy.

#### Data processing and analysis

The data were entered into EpiData version3.1 software and was exported to STATA version14 software for analysis. Tobit model was used to estimate and identify determinants of willingness to pay. Tobit model is used to estimate outcome variables from censored data (skewed distribution of data). This model also predicts maximum amount of money that individuals were willing to pay for human papilloma virus vaccine. This model has an advantage over logistic discrete choice model in that it reveals the maximum amount of money the respondents were willing to pay.

$$ y = \left\{ {\begin{array}{*{20}c} \begin{aligned} &{1\quad {\text{i}}.\,{\text{MWTP}} = \beta {\text{o}} + \beta^{\prime}\, {\text{Xi}} + {\text{e}} > 0} \\ &{0\quad {\text{if MWTP}} \le 0} \\ \end {aligned} \end{array} } \right. $$

where, Y = outcome variable; MWTP = maximum WTP; Xi = explanatory variables; βo = slope; β′ = coefficient; e = error term; 1 = success/yes; 0 = failure/no. p-value = 0.05 was used to determine statistical significance.

### Results

#### Socio-demographic and economic, and health status and services related characteristics of the respondents

A total of 392 study participants, with a response rate of 92.7%, were included in the study. The mean age of the respondents was 28 years. Majority of respondents were Orthodox, Amhara, single, first degree holders and nurses (Table 1). Average monthly income of participants was 6177.47ETB. Around 57.4% of respondents had high perceived quality of the HPV vaccine service and 56.63% of respondents said they had a good health status (Table 1).

**Table 1:** Demographic, socioeconomic and health status and services related characteristics of study participants at CMHS, 2018

Variable	Description	Frequency (%)
Religion of the respondent	Orthodox	318 (81.33%)
	Muslim	53 (13.55%)
	Protestant	17 (4.35%)
	Others	3 (0.77%)
Ethnicity of the respondent	Amhara	347 (88.52%)
	Tigre	24 (6.12%)
	Oromo	10 (2.55%)
	Others	11 (2.81%)
Marital status of the respondent	Single	242 (61.73%)
	Married	136 (34.69%)
	Divorced	11 (2.81%)
	Widowed	3 (0.77%)
Background professional specialities of the respondent	Nurse	185 (47.19%)
	Midwifery	79 (20.15%)
	Public health officer	45 (11.48%)
	Pharmacy	44 (11.22%)
	Medical laboratory	23 (5.87%)
	Others	16 (4.08%)
Educational status of the respondent	Diploma	73 (18.62%)
	First degree	277 (70.66%)
	Second degree	42 (10.71%)
Health status of participants (n = 392)	Poor	34 (8.67%)
	Medium	136 (34.69%)
	Good	222 (56.63%)
Perceived quality of the service (n = 392)	Very low	12 (3.06%)
	Low	60 (15.31%)
	Medium	42 (10.71%)
	High	225 (57.40%)
	Very high	53 (13.52%)

#### Health professionals’ knowledge on cervical cancer and its risk factors

In this study 97.96% of respondents had heard about human papilloma virus vaccine. Schools were the main means of information about HPV vaccine for 60.68% respondents. Among the respondents 68.62% had excellent knowledge about cervical cancer danger signs and its risk factors (Table 2).

**Table 2:** Health professionals’ knowledge about cervical cancer and its risk factors at CMHS 2018

Variable	Description	Frequency %
Do you have heard about human papilloma virus vaccine? (n = 392)	Yes	384 (97.96%)
	No	8 (2.04%)
What is your source of more information (n = 384)	School	233 (60.68%)
	TV/Radio	101 (26.30%)
	Training	33 (8.59%)
	Internet	14 (3.65%)
	Other	3 (0.78%)
Perceived seriousness of cervical cancer (392)	Less serious	52 (13.27%)
	Serious	227 (57.91%)
	Highly serious	113 (28.83%)
Knowledge of respondents (n = 392)	Poor knowledge	27 (6.89%)
	Moderate knowledge	96 (24.49%)
	Excellent knowledge	269 (68.62%)

#### Willingness to pay (WTP) for HPV service

Most of respondents (85.97%) were willing to pay for human papilloma virus vaccination. Among participants 56.37% of them were willing to pay more than 200ETB and 31.63% were willing to pay more than 300ETB. The mean (± SD) amount of money health professionals willing to pay was 231.34ETB ± 132 per visit or 7.16 $USD.

It’s calculated as follows:

$$ \begin{aligned} {\text{AMWTP}} = & \mathop \sum \limits_{{{\text{n}} = 1}}^{392} \left( {{\text{MWTP}}1 + {\text{MWTP}}2 + \cdots \ldots \ldots .. + {\text{MWTP}}545} \right) \div 392 \\ = 2 3 1. 3 4 {\text{ETB per HPV vaccination service}} \\ \end{aligned} $$

Currency exchange in March 2018 was 1$USD = 27.2ETB.

#### Factors associated with willingness to pay for HPV to prevent cervical cancer

The study showed that age, educational status, knowledge about cervical cancer and its risk factors and monthly income were factors statistically associated with willingness to pay for HPV vaccination in the multivariable analysis of Tobit model at a p-value ≤ 0.05 (Table 3).

**Table 3:** Factors associated with WTP for human papilloma virus vaccination in CMHS, University of Gondar, 2018

Parameter for MWTP	Category	Coefficient	Standard error	t-value	p-value	95% confidence interval
Age	N	− 2.4	1.2	− 2.00	*0.047*	− 4.8, − 0.04
Marital status (ref. married)	S					
Single		− 6.8	12.1	− 0.57	0.572	− 30.6, 16.9
Divorced		− 4.4	35.1	− 0.13	0.899	− 73.4, 64.5
Widowed		19.7	56.1	0.35	0.726	− 90.8, 130.2
Background profession (ref. public health officer)	S					
Pharmacy		17.6	21.5	0.82	0.414	− 24.8, 60
Nurse		− 12.8	17.3	− 0.74	0.461	− 46.8, 21.3
Midwifery		− 7.9	19.5	− 0.40	0.686	− 46.3, 30.5
Medical laboratory		− 2.4	29.3	− 0.08	0.935	− 60.1, 55.3
Others		− 38.5	27.6	− 1.40	0.164	− 92.8, 15.8
Educational status (ref. Diploma)	S					
First degree		− 63	19	− 3.32	*0.001*	− 100.4, − 25.7
Second degree		− 10.5	26.9	− 0.39	0.697	− 63.4, 42.4
Knowledge (ref. poor knowledge)	S					
Moderate knowledge		56.8	26.1	2.18	*0.030*	5.6, 108.1
Excellent knowledge		69.7	24.3	2.87	*0.004*	22, 117.5
Health status (ref. poor	S					
Medium		− 24.6	21.9	− 1.12	0.262	− 67.6, 18.4
Good		− 18.6	20.6	− 0.90	0.367	− 59, 21.9
Perceived seriousness cervical cancer (ref. less serious)	S					
Serious		− 35	17.9	− 1.95	0.053	− 70.4, 0.4
Highly serious		− 21.3	19.6	− 1.09	0.278	− 59.8, 17.3
Monthly income (Birr)	N					
		0.03	0.004	8.77	0.000***	0.03, 0.04

Italic values indicate statistically significant associations

*S* string variable, *N* numeric value and *D* Dummy variable

* Significant with p-value ≤ 0.05

*** Significant with p-value ≤ 0.001

As age increased by 1 year, respondents were willing to pay decreased by 2.5 Birr for human papilloma virus vaccination by holding other variables constant (Table 3).

First degree holders were willing to pay 63ETB less than those diploma holders for HPV vaccine by keeping other variables constant (Table 3).

The respondents who had excellent knowledge about cervical cancer were willing to pay 69.7ETB more than those who had poor knowledge for human papilloma virus vaccine by keeping other variables constant (Table 3).

As monthly income of the respondents increased by 1ETB, the willingness to pay increased by 0.04ETB for human papilloma virus vaccination by holding other conditions constant (Table 3).

### Discussion

The finding of the present study showed that age, educational status, knowledge about cervical cancer and its risk factors and monthly income were significant determinants of willingness to pay for human papilloma virus vaccination.

This study revealed that 85.97% were willing to pay for HPV service. The result was line with studies done in Arba Minch town in 2011 on willingness to pay for insecticide treated bed net (86%) [12]. On the other side, this study showed that willing to pay health professionals for HPV were higher than willing to pay for injectable contraceptive and foot wear for podoconiosis which were (68%) and (72.8%) respectively, in studies done in Tigray and northern Ethiopia [13, 14]. This may be due to study population difference which may not have equal information about health. However, the result of the study was lower than willingness of respondents to pay for health service in Iran which was 88.8% [15]. The possible reason might be difference in study areas, cultural and demographic and study population differences.

The average amount of money, the respondents willing to pay for HPV, was 231.34ETB (US$8.51) which was greater than the price used to elicit preference by 31ETB. The amount of money respondents were willing to pay for HPV was much higher than the amount of money respondents were willing to pay for injectable contraceptive per service and footwear for podoconiosis per year which were 11 Birr (US$0.65) and 64 Birr (US$ 3.28) [13, 14]. The possible explanation for this may be study population difference, the currency inflation and program differences. But the result of the study was lower than a study done on willingness to pay for HPV vaccine which was US$ 11.68 in Nigeria [16] and US$ 50.26 in Malaysia [17], and WTP for the clinical preventive services which was US$93.72 in South-Nigeria [18]. This may be due to the difference in socioeconomic difference of the participants like per capita income, study population difference.

The age of respondents had negatively associated with willingness to pay. The negative association may be as age respondents’ increase, they may have less family income due to large family than younger age groups might be the possible explanations. This result was inconsistent with studies done on WTP for Pap test in Nigeria [19], willingness to pay for health care services in Malaysia [20] and Iran [21] had a positive association with WTP. The possible difference might be different per capital income of those countries. This result contradicted with studies done on WTP for mortality risk reduction from the environment in USA [22] which reveals age does not have association with WTP for mortality risk reduction. One possible explanation for this difference may be all age groups equally risky by environmental risk factors in studies done USA.

This study also showed that educational status was identified as significant variable to WTP for HPV. Possibly this might be as educational status increases, individuals might have more information and aware about their health issues. This report was consistent with studies done Addis Ababa [23], Malaysia [20], Iran [21], Arba Minch [12] which revealed educational status had positive association with the preference and utilization of different health services.

The knowledge of health professionals’ about cervical cancer and its risk factors had statistical association with willingness to pay. The possible explanation for this might be as individuals know more about cervical cancer and its risk factors, they become more willing to prevent cervical cancer. This finding was consistent with other studies conducted in Ethiopia [24], USA [25] which showed association of knowledge with the preference and utilization of different health services.

The monthly income of the respondents’ had significant association WTP for human papilloma virus vaccination. This may be due to those having more income may had additional money to be allocated for promotion of their health in addition to basic needs. Studies conducted in Addis Ababa [23], Malaysia [20] Iran [15], Korea [26] and USA [27] for different health care services supported this evidence.

### Conclusion and recommendation

This study indicated that majority of the health professionals were willing to pay for HPV Vaccination service. Age, educational status, knowledge about cervical cancer and its risk factors, and monthly income of the respondents were statistically associated with willingness to pay for HPV service.

Policy makers shall consider human papilloma virus vaccine to prevent cervical cancer maintain health of women’s and do more on rising awareness of individuals about cervical cancer and its risk factors.

## Limitation of the study

The method used in this study is partial economic evaluation approach which it did not account other cervical cancer prevention methods. The study only includes educated female health professional might be the limitation of this study. Another limitation of this study was respondents’ bias that aims to reduce the cost of payment to the service and those have family history of cervical cancer need HPV vaccination might overestimate the cost.

## Abbreviations

AMWTP: average maximum willingness to pay
CHMS: College of Medicine and Health Sciences
CV: Contingent Valuation
ETB: Ethiopian Birr
HPV: human papilloma virus vaccine
MWTP: maximum willingness to pay
UoGSRH: University of Gondar Specialized Referral Hospital
USA: United Nation of America
USD: United Nation Dollar
WTP: willingness to pay
